# Respiratory virus surveillance in Canada during the COVID-19 pandemic: An epidemiological analysis of the effectiveness of pandemic-related public health measures in reducing seasonal respiratory viruses test positivity

**DOI:** 10.1371/journal.pone.0253451

**Published:** 2021-06-18

**Authors:** Kyu Young Park, Sumin Seo, Junhee Han, Ji Young Park

**Affiliations:** 1 Faculty of Dentistry, University of British Columbia, Vancouver, British Columbia, Canada; 2 Division of Biostatistics, Hallym Institute for Clinical Medicine, Hallym University Medical Center, Anyang, Republic of Korea; 3 Department of Statistics, Hallym University, Chuncheon, Republic of Korea; 4 Department of Pulmonary, Allergy and Critical Care Medicine, Hallym University Sacred Heart Hospital, Anyang, Republic of Korea; Columbia University, UNITED STATES

## Abstract

**Background:**

Various public health measures have been implemented globally to counter the coronavirus disease 2019 (COVID-19) pandemic. The purpose of this study was to evaluate respiratory virus surveillance data to determine the effectiveness of such interventions in reducing transmission of seasonal respiratory viruses.

**Method:**

We retrospectively analysed data from the Respiratory Virus Detection Surveillance System in Canada, before and during the COVID-19 pandemic, by interrupted time series regression.

**Results:**

The national level of infection with seasonal respiratory viruses, which generally does not necessitate quarantine or contact screening, was greatly reduced after Canada imposed physical distancing and other quarantine measures. The 2019–2020 influenza season ended earlier than it did in the previous year. The influenza virus was replaced by rhinovirus/enterovirus or parainfluenza virus in the previous year, with the overall test positivity remaining at approximately 35%. However, during the 2019–2020 post-influenza period, the overall test positivity of respiratory viruses during the COVID-19 was still low (7.2%). Moreover, the 2020–2021 influenza season had not occurred by the end of February 2021.

**Conclusion:**

Respiratory virus surveillance data may provide real-world evidence of the effectiveness of implemented public health interventions during the current and future pandemics.

## Introduction

In December 2019, severe acute respiratory syndrome coronavirus 2 (SARS-CoV-2) was first identified in clusters of pneumonia cases of unknown aetiology in Wuhan, China [1]. The SARS-CoV-2 spread rate has increased exponentially worldwide. The first coronavirus disease (COVID-19) case in Canada was diagnosed on 25 January 2020 [2, 3]. Nearly every country has employed a variety of infection control measures; however, the degree of control varied between countries [4]. To attain an optimal balance of public health policies, the effectiveness of public health interventions against COVID-19 needs to be evaluated [5]. As the level of SARS-CoV-2 dissemination in each community differed when public health measures were first implemented, the same policies and assumptions of compliance yielded varying levels of success in infection control. In particular, since there was no herd immunity against SARS-CoV-2, public health interventions alone were insufficient to control the pandemic in most communities [6, 7]. However, this cannot be considered direct evidence that these measures are invalid [8].

Seasonal respiratory viruses have long periods of cyclic oscillation in communities residing in temperate areas [9]. Different respiratory viruses spread sequentially according to seasonal changes in temperature and humidity in a predictable manner every year [10, 11]. A similar pattern of cyclic oscillation has been observed in countries with a temperate climates where the COVID-19 pandemic is severe [9]. Other important determinants of the seasonality of viruses are host behaviour patterns (e.g. staying indoors) and host susceptibility (e.g. susceptible children attending school), which are also affected by seasonal climate changes [12]. On the other hand, outside healthcare settings, unless a severe epidemic occurs, patients with common colds or acute lower respiratory tract infections relayed to these seasonal respiratory viruses are neither quarantined nor undergo contact screening. Prevention and control recommendations have been put forth to control nosocomial or long-term residential care infections, including influenza.

We conducted our study against this background, using national public health surveillance data to evaluate the effectiveness of public health measures, recommended in response to the COVID-19 pandemic, against transmission of seasonal respiratory viruses in Canada.

## Methods

### Canada’s response to COVID-19

The World Health Organization declared the global outbreak of COVID-19 a pandemic on 11 March 2020. Table 1 shows the timeline of the initiation of public health measures implemented in Canada [13, 14]. Orders to stay at home were recommended to the public and the importance of applying physical distancing was emphasised [15]. On 18 March, foreign travellers were banned from entering Canada. During this period, closure of public facilities such as schools, religious facilities, and libraries was ordered by provincial and territorial governments [2, 15]. From the end of March, a more intense social distancing policy was implemented, and all non-essential businesses were closed. The Canadian government provided financial help to maintain social distancing and to support vulnerable Canadians through the COVID-19 Economic Response Plan [2]. The Public Health Agency of Canada educated the public about physical distancing, hand hygiene, and cleaning with disinfectants. Anyone with symptoms of COVID-19 was asked to self-isolate for 14 days. In situations where physical distancing was not possible, such as when making use of public transportation, non-medical masks or face coverings were recommended as complementary to the above measures.

**Table 1:** Timeline of the initiation of public health measures implemented in Canada.

Date of initiation	Public health measures
12 March 2020	Cancellation of public events
14 March 2020	Stay-at-home orders
16 March 2020	School closures
16 March 2020	Restrictions on gathering size (2–50, vary between provinces or territories)
18 March 2020	Workplace closures
18 March 2020	International travel restrictions (total border closure)
20 March 2020	Internal movement restrictions (limit non-essential travel to other provinces or territories)
20 May 2020	Official mask-wearing recommendation
7 July 2020	Mandatory use of masks or face covering in enclosed public spaces

### Data sources of virus surveillance

We analysed surveillance data released by the Canadian health authorities and the Centre for Immunization and Respiratory Infectious Diseases [16, 17]. They reported the weekly incidence of influenza as well as surveillance results of other respiratory viruses from laboratories and hospitals across Canada, including respiratory syncytial virus (RSV), human rhinovirus/enterovirus (RV/EV), adenovirus (ADV), seasonal human coronavirus (COV), human metapneumovirus (MPV), parainfluenza virus (PIV), and influenza A/B (IFV) [18]. SARS-CoV2 was not included in this surveillance. Respiratory virus testing is generally a part of free universal healthcare in Canada and is implemented on a national scale; therefore, the surveillance programme provides real-world, scaled, and comprehensive data [17]. These surveillance data are updated weekly and available to the public. These results are not from data collected in a centralised laboratory, and different multiplex polymerase chain reaction (PCR) panels for detecting viruses were used in the respective laboratories [19].

### Comparison of seasonal respiratory viral epidemics

This study compared the epidemic patterns of respiratory viruses over the past 5 years, including those during the COVID-19 pandemic. We compared changes in test positivity of individual and overall respiratory viruses over time in Canada. An interrupted time series analysis was conducted to estimate the effectiveness of the pandemic-related interventions against the transmission of seasonal respiratory viruses. The starting point of the intervention for was defined as the 11^th^ week of 2020, for analysis. The early stages of the COVID-19 pandemic overlapped with the influenza epidemic period in countries with temperate climates in the northern hemisphere, including Canada. For this reason, it is worth analysing changes in the influenza epidemic pattern. In the current study, the influenza epidemic period was determined using laboratory surveillance data, starting with the week before the threshold value of influenza test positivity (2.5%) was reached, and ending with the week after test positivity decreased below 2.5%. As for the seasonal influenza epidemic, IFV types A and B occur sequentially or simultaneously each year. In this study, the results of influenza A were used to define the duration of the seasonal influenza epidemic. The durations of exacerbation and relief in the seasonal epidemics were compared, as well as the rate of remission. Furthermore, we compared the test positivity of all tested respiratory viruses present during the inter-influenza epidemic periods with that of other years.

### Statistical analysis

Data were expressed as the total number of tests and test positivity for individual viruses by week. Segmented regression analysis of interrupted time series data is useful for evaluating the effect of intervention over time, even when randomization is not possible or control groups cannot be set [20]. We analysed weekly aggregated respiratory virus test positivity (time series data) using segmented regression analysis. The time segments were divided into two periods: before and after the intervention (public health measures). The changes in levels (baseline value of the outcome at time zero) and trends (the rate of change) of the pre-intervention segment and post-intervention segment were analysed. The statistical model was specified as follows [21]:

$$Y_{t}=\gamma_{o}+\gamma_{1}*preslope+\gamma_{2}*intervention+\gamma_{3}*postslope+\varepsilon_{t}$$

Y_t_ is the outcome variable (test positivity for specific virus) at time t. *γ*_*0*_ implies the baseline level at the first analysis (time zero). *γ*_*1*_ estimates the trend of changes in the virus test positivity before the intervention. *Preslope* is a continuous variable, coded sequentially from 1 (first data analysis) until the introduction of the intervention. *γ*_*2*_ represents the change in test positivity levels immediately after the intervention. *Intervention* is a dichotomous variable equal to 0 before the intervention and 1 after the intervention. Estimate *γ*_*3*_ captures the trend after the intervention. *Postslope* is coded as 0 up to the intervention and sequentially coded from 1 after that point. The stationarity and seasonality were evaluated by decomposing the time series data. When seasonality was confirmed, further analysis was performed at a seasonally adjusted test positivity, and generalized least squares (GLS) was performed to compare the effects of the intervention in consideration of data non-stationarity and autocorrelation. The autocorrelation function (ACF) and partial autocorrelation function (PACF) diagrams were depicted, and the autoregressive–moving-average (ARMA; p, q) model was selected, incorporating the extended sample autocorrelation function (EACF). The final model was selected as that with the lowest Akaike Information Criterion (AIC) and Bayesian Information Criterion (BIC) indices. We used R statistical software version 4.0.2 (R Foundation for Statistical Computing, Vienna, Austria) for all analyses. A p-value < 0.01 was considered statistically significant. This study was a secondary analysis of de-identified and publicly available data. For this reason, it did not require ethics committee approval.

## Results

Between the 35^th^ week of 2015 and the 3^rd^ week of 2021, 1,411,247 influenza test results and a mean of 729,639 test results for other respiratory viruses were collected (Table 2). From the 12^th^ week of 2020 to the 3^rd^ week of 2021 (after intervention), 484,780 tests for influenza and a mean of 297,226 tests for other respiratory viruses were collected (RSV 442,047; PIV 283,425; ADV 282,223; MPV 283,326; RV/EV 278,286; COV 214,050).

**Table 2:** The number of tests for individual respiratory viruses in Canada.

Year	Duration	Number of tests for individual respiratory viruses						
		RSV	PIV	ADV	MPV	RV/EV	COV	IFV
2015	18 weeks	48774	37968	38056	35195	21404	24505	53164
2016	52 weeks	231478	151942	152450	142227	95638	107584	260615
2017	52 weeks	250820	154767	154680	149412	114156	131402	276028
2018	52 weeks	301231	152999	150307	146189	90148	108743	332465
2019	52 weeks	296996	145946	145131	138258	93125	107956	312708
2020	53 weeks	576962	336871	335245	336263	322810	255005	617535
2021	3 weeks	29398	10346	10347	10173	9937	8347	43512

Respiratory syncytial virus (RSV), parainfluenza virus (PIV), adenovirus (ADV), human metapneumovirus (MPV), human rhinovirus/enterovirus (RV/EV), seasonal human coronavirus (COV), and influenza A/B (IFV).

### Change in seasonal respiratory viral epidemics

**Fig 1:**
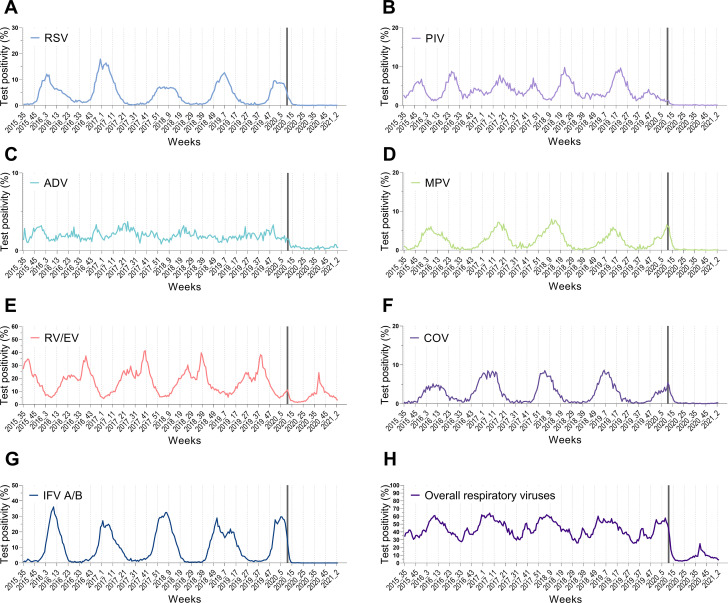
Test positivity of respiratory viruses based on weekly laboratory surveillance data in Canada. The grey vertical bar indicates initiation of preventive measures against coronavirus disease 2019. Respiratory syncytial virus (RSV), parainfluenza virus (PIV), adenovirus (ADV), human metapneumovirus (MPV), human rhinovirus/enterovirus (RV/EV), seasonal human coronavirus (COV), and influenza A/B (IFV).

Table 3 shows the results of interrupted time series regression of the test positivity of each respiratory virus (Fig 1). Before the public health interventions, there were no significant changes in trend among all respiratory viruses (γ_1_). After the intervention, the test positivity for RSV, PIV, MPV, COV, and IFV decreased with statistical significance (γ_3_). On the other hand, for ADV and RV/EV, the decrease was not statistically significant (Table 3). In regression analysis of the test positivity of overall respiratory viruses, the seasonality and trend were confirmed by decomposing the weekly time series data. Autocorrelation was confirmed by evaluating the ACF and PACF graphs of adjusted overall test positivity, which removed the influence of seasonality. The ACF slowly attenuated to zero, and the PACF decreased after 1; therefore, the AR (1) model was considered, and the ARMA (1,1), ARMA (1,2), and ARMA (2,1) models were considered in the EACF table. Among them, ARMA (1,0), which had the lowest AIC and BIC, was selected as the final model. After the intervention, the trend (γ_3_) was calculated as -0.946 (p < 0.001), and it was confirmed that the test positivity for overall respiratory viruses had decreased after the public health interventions. The detailed results of the analyses of other viruses are presented in the S1 File.

**Table 3:** Segmented regression analysis of the test positivity of respiratory viruses.

Respiratory viruses	Final ARMA models	*Intercept (γ0)*	*Preslope (γ1)*	*Intervention (γ2)*	*Postslope (γ3)*	p-value (*γ3*)
RSV	ARMA (2,1)	5.066	-0.005	-0.410	-0.134	p = 0.001
PIV	ARMA (2,2)	4.684	-0.007	0.017	-0.076	p = 0.013
ADV	ARMA (1,1)	2.174	-0.002	-0.711	-0.015	p = 0.145
MPV	ARMA (2,2)	2.818	-0.003	0.068	-0.053	p = 0.028
RV/EV	ARMA (1,0)	19.981	-0.023	-1.440	-0.125	p = 0.226
COV	ARMA (1,1)	3.169	-0.005	1.846	-0.131	p<0.001
IFV	ARMA (3,0)	9.314	0.006	1.613	-0.388	p<0.001
Overall	ARMA (1,0)	46.996	-0.035	-1.835	-0.946	p<0.001

Autoregressive–moving-average (ARMA; p, q), Respiratory syncytial virus (RSV), parainfluenza virus (PIV), adenovirus (ADV), human metapneumovirus (MPV), human rhinovirus/enterovirus (RV/EV), seasonal human coronavirus (COV), and influenza A/B (IFV).

### Change in seasonal influenza epidemics

**Fig 2:**
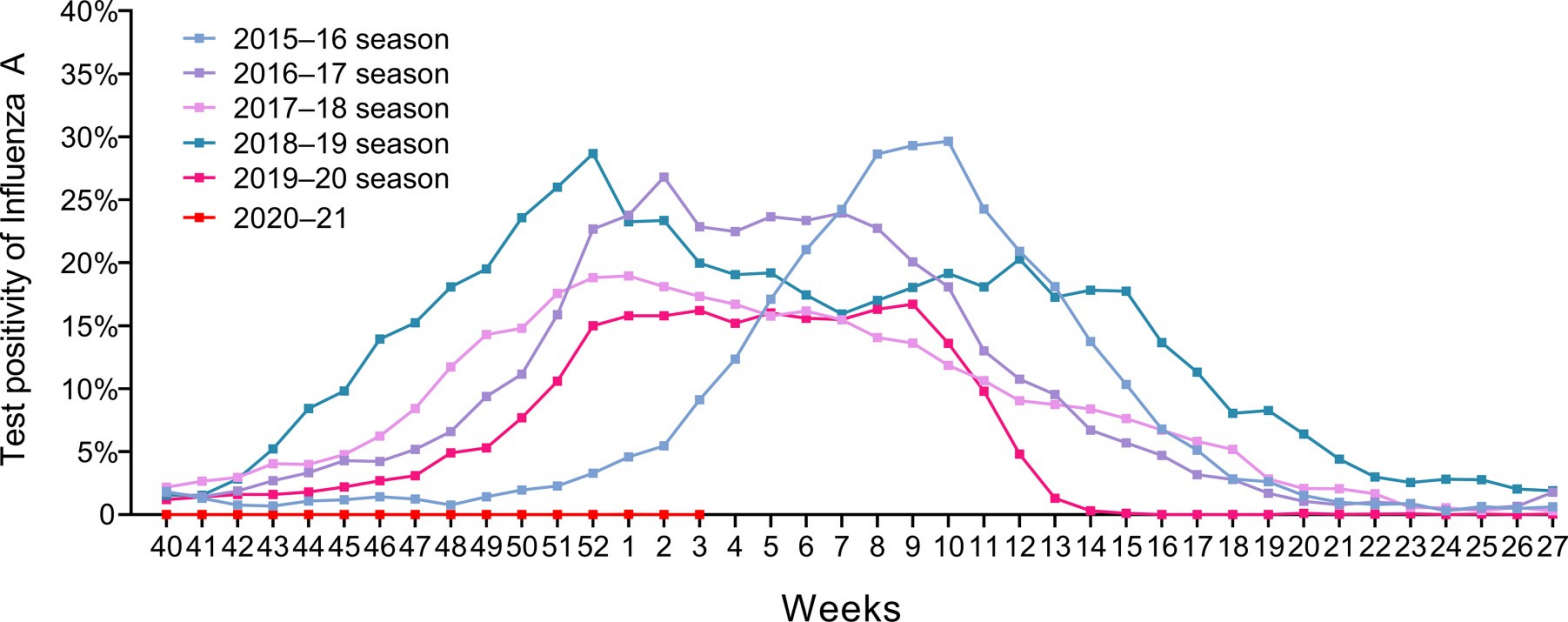
Laboratory-confirmed influenza A epidemic curves in Canada by year.

The epidemic period of the 2019–2020 influenza A season was 19 weeks, which is numerically shorter compared to previous seasons. In particular, the relief period from the peak of influenza A positivity to the end of the epidemic was shortened to 3 weeks (Fig 2 and Table 4). In comparison, the relief periods for the previous four seasons were much longer (2018–2019: 25 weeks; 2017–2018: 18 weeks; 2016–2017: 11 weeks; 2015–2016: 9 weeks). Moreover, the 2020–2021 influenza epidemic had not occurred by February 2021 [19].

**Table 4:** Analysis of annual influenza A incidence rate confirmed by laboratory testing.

	Years of influenza A season				
Variable	2015–2016	2016–2017	2017–2018	2018–2019	2019–2020
Duration of seasonal epidemics, weeks	20	28	33	36	19
Duration of epidemic phases, weeks*					
Exacerbation phases	11	17	15	11	16
Relief phases	9	11	18	25	3
Alleviation rates of seasonal epidemics					
Mean reduction in weekly test positivity rate	-3.00%	- 1.92%	- 0.90%	- 1.04%	- 3.97%

*Statistical significance was tested by linear-by-linear association (p = 0.003).

### Respiratory viral epidemics between influenza epidemic periods

**Fig 3:**
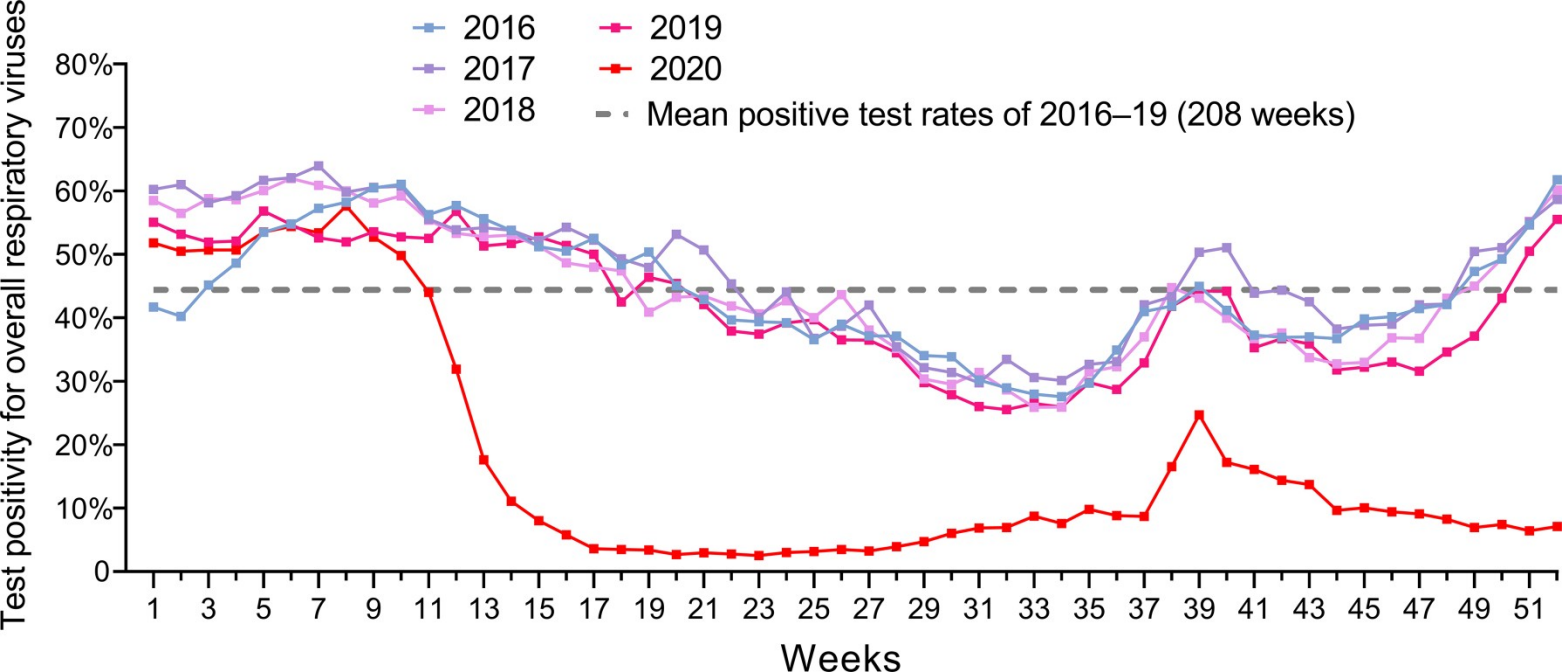
Annual test positivity of overall respiratory viruses based on laboratory surveillance in Canada for every week during 2016–2020.

**Fig 4:**
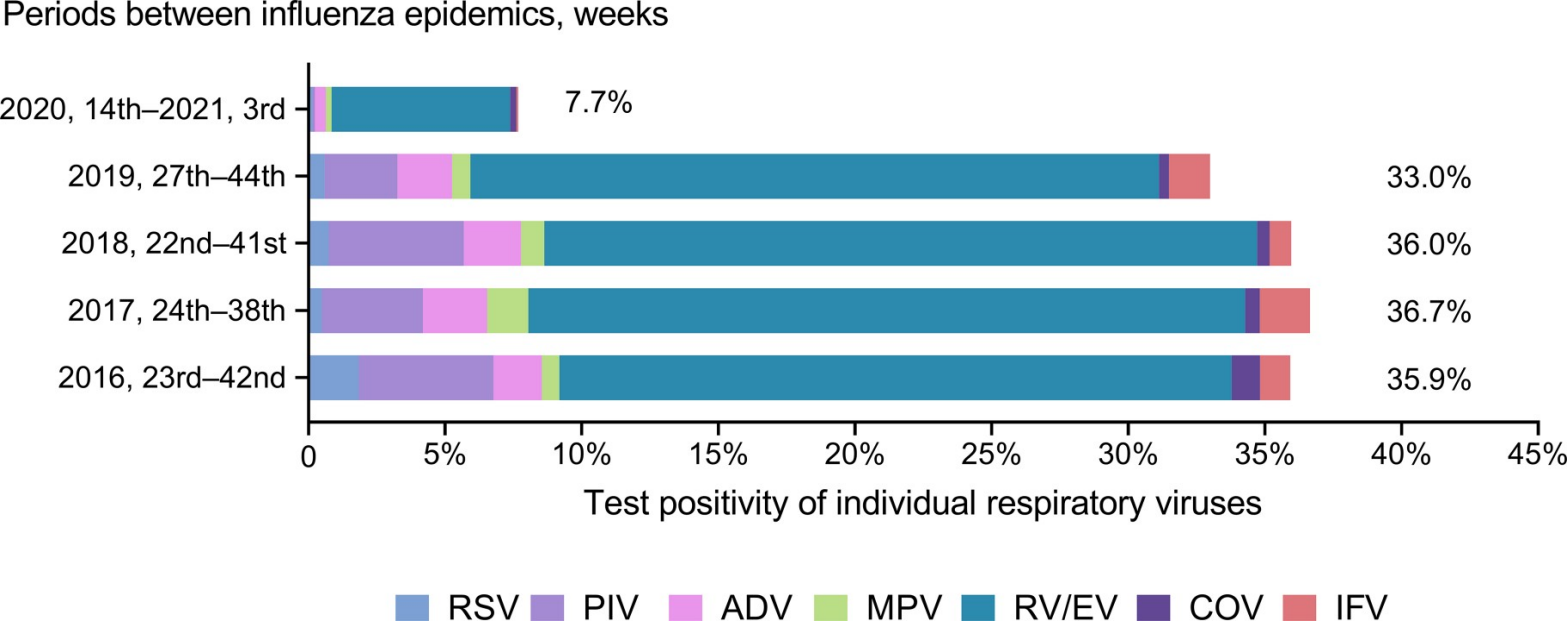
Test positivity for individual respiratory viruses using laboratory surveillance stratified by the periods between influenza epidemics. Respiratory syncytial virus (RSV), parainfluenza virus (PIV), adenovirus (ADV), human metapneumovirus (MPV), human rhinovirus/enterovirus (RV/EV), seasonal human coronavirus (COV), and influenza A/B (IFV).

The overall respiratory virus test positivity during the influenza epidemic and post-epidemic periods for five consecutive seasons are shown in Fig 3. Between influenza epidemic periods, the overall test positivity was lower in 2020 than in previous years. In previous years, RV/EV or PIV became prevalent after the influenza epidemic. After the 2019–2020 season, the test positivity of these viruses continued to be very low (Fig 4).

## Discussion

The measures implemented because of the COVID-19 pandemic were effective in controlling seasonal respiratory viral transmission in communities that did not undergo quarantine or contact screening.

As the number of confirmed COVID-19 cases is based on PCR tests, there is a risk of underestimation [22, 23]. The results of COVID-19 sero-epidemiologic surveys using antibody tests showed much higher infection rates than did those based on reported cases [24, 25]. Among communities, there is a significant difference in the availability of COVID-19 tests and indications for testing. The number of confirmed patients undergoing symptomatic examination may be subject to selection bias. In fact, there are many differences in the actual COVID-19 mortality rates among developed European countries [26]. For these reasons, assessing the effectiveness of public health measures with the number of confirmed COVID-19 cases is challenging and should be interpreted with care. Therefore, seasonal respiratory virus surveillance data may be used as a complementary tool to assess compliance with and the effectiveness of public health measures.

Consistent with previous studies’ results [17], our results demonstrated seasonality and a similar, cyclic pattern of influenza epidemics every year. Individual viruses exhibited sequential epidemic patterns, and the overall test positivity rate maintained a similar pattern every year. However, after applying physical distancing and preventive measures in response to the COVID-19 outbreak, there were large changes in the epidemic patterns of respiratory viruses. Since there was no application of additional vaccines or the introduction of new therapeutic agents for seasonal viruses, it is not unreasonable to assume that the pandemic-related interventions caused these changes. These favourable effects have been reported in previous studies, although those analyses were limited to influenza or reports of small-scale surveillance data without time-series analysis [27, 28]. Our study’s advantage was in the analysis of national data of respiratory virus infections in Canada, incorporating a well-designed reporting system from local health authorities. Besides, since long-term data were available for analysis, changes in trends following public health interventions could be evaluated after appropriately excluding seasonality.

One of the limitations of this study is that the specific preventive intervention (e.g., physical distancing vs. face masking) with the greatest effectiveness in controlling the transmission of viral respiratory diseases in Canada could not be determined. Comparisons and analyses of quarantine measures adopted by various countries may provide better clarity. Another limitation is that changes in medical behaviour following the onset of the COVID-19 pandemic might have affected the results. However, in this study, we used test positivity rather than patient numbers for analyses, thereby showing that the actual number of patients with respiratory infections decreased. Moreover, during the COVID-19 epidemic, more test results were collected than during the same period in previous years. Lastly, we analysed surveillance data from all over Canada but did not stratify the testing performance geographically to confirm a similar population density of testing across Canada. We assumed that the testing density would be identical in all the provinces since the medical staff use their discretion in testing the patients consulting at medical institutions for acute respiratory symptoms.

## Conclusions

Our study demonstrated the effectiveness of COVID-19-related public health interventions against the transmission of seasonal respiratory viruses in a real-world setting. These results may be used as evidence for the early adoption of appropriate public health measures against future infectious respiratory diseases.

## Supporting information

S1 File The detailed results of the analyses of other viruses. (DOCX)

## List of abbreviations

ADV: adenovirus
COV: coronavirus
COVID-19: coronavirus disease 2019
IFV: influenza A/B virus
MPV: human metapneumovirus
PCR: polymerase chain reaction
PIV: parainfluenza virus
RSV: respiratory syncytial virus
RV/EV: human rhinovirus/enterovirus
SARS-CoV-2: severe acute respiratory syndrome coronavirus 2
