# Supplementary material for: Respiratory virus surveillance in Canada during the COVID-19 pandemic: An epidemiological analysis of the effectiveness of pandemic-related public health measures in reducing seasonal respiratory viruses test positivity
Source: PLoS One. 2021 Jun 18;16(6):e0253451. doi: 10.1371/journal.pone.0253451 (PMC8213179; doi:10.1371/journal.pone.0253451)
Supplement: S1 File — (DOCX) [file pone.0253451.s001.docx]

**Respiratory virus surveillance in Canada during the COVID-19 pandemic: an epidemiological analysis of the effectiveness of pandemic-related public health measures in reducing seasonal respiratory viruses test positivity**

Kyu Young Park, Sumin Seo, Junhee Han, and Ji Young Park

**Abbreviation**

Respiratory syncytial virus (RSV)

Human rhinovirus/enterovirus (RV/EV)

Adenovirus (ADV)

Seasonal human coronavirus (COV)

Human metapneumovirus (MPV)

Parainfluenza virus (PIV)

Influenza A/B (IFV)

Autocorrelation function (ACF)

Partial autocorrelation function (PACF)

Akaike Information Criterion (AIC)

Bayesian Information Criterion (BIC)

**S1 Table.** Weekly reported test positivity of overall respiratory viruses (A), decomposition of the time series of overall respiratory viruses (B), autocorrelation function of adjusted test positivity (C), and partial autocorrelation of adjusted test positivity of overall respiratory viruses (D).


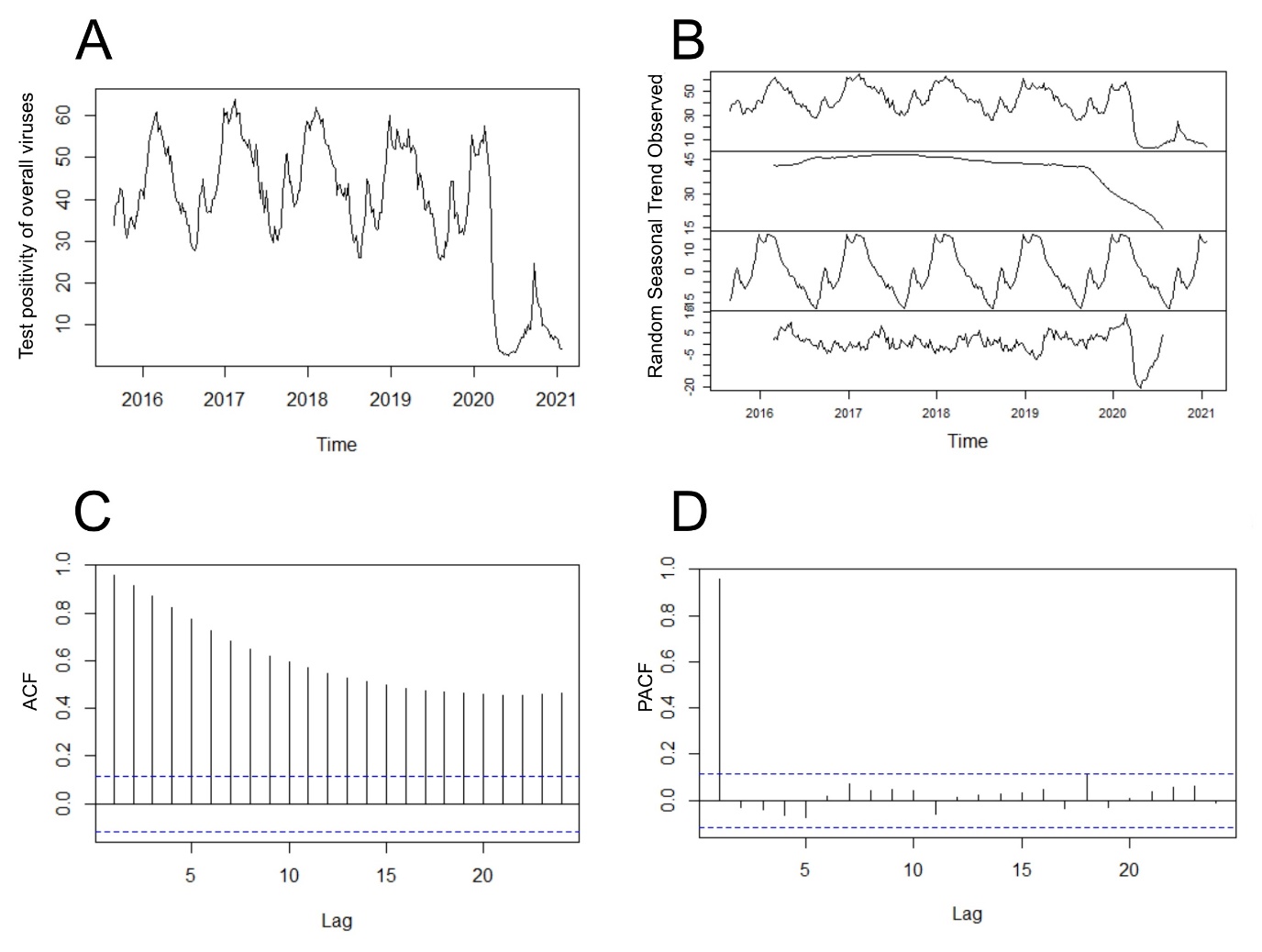


**S2 Table.** Segmented linear regression of overall respiratory virus test positivity.

|  | ARMA (1,0)* | | ARMA (1,1) | | ARMA (1,2) | | ARMA (2,1) | |
| --- | --- | --- | --- | --- | --- | --- | --- | --- |
|  | Coefficient | P-value | Coefficient | P-value | Coefficient | P-value | Coefficient | P-value |
| Intercept | 46.996 | <0.001 | 46.987 | <0.001 | 47.067 | <0.001 | 47.005 | <0.001 |
| Preslope | -0.035 | 0.273 | -0.035 | 0.269 | -0.036 | 0.247 | -0.035 | 0.274 |
| Intervention | -1.835 | 0.469 | -1.618 | 0.524 | -0.339 | 0.893 | -1.805 | 0.476 |
| Postslope | -0.946 | <0.001 | -0.950 | <0.001 | -0.979 | <0.001 | -0.947 | <0.001 |
| AIC | 1322.64 | | 1324.51 | | 1323.31 | | 1326.48 | |
| BIC | 1344.49 | | 1350.00 | | 1352.45 | | 1355.61 | |

*The final model

**S3 Table.** Weekly reported test positivity of influenza A and B viruses (A), decomposition of the time series of influenza viruses (B), autocorrelation function of adjusted test positivity of influenza viruses (C), and partial autocorrelation of adjusted test positivity of influenza viruses (D).


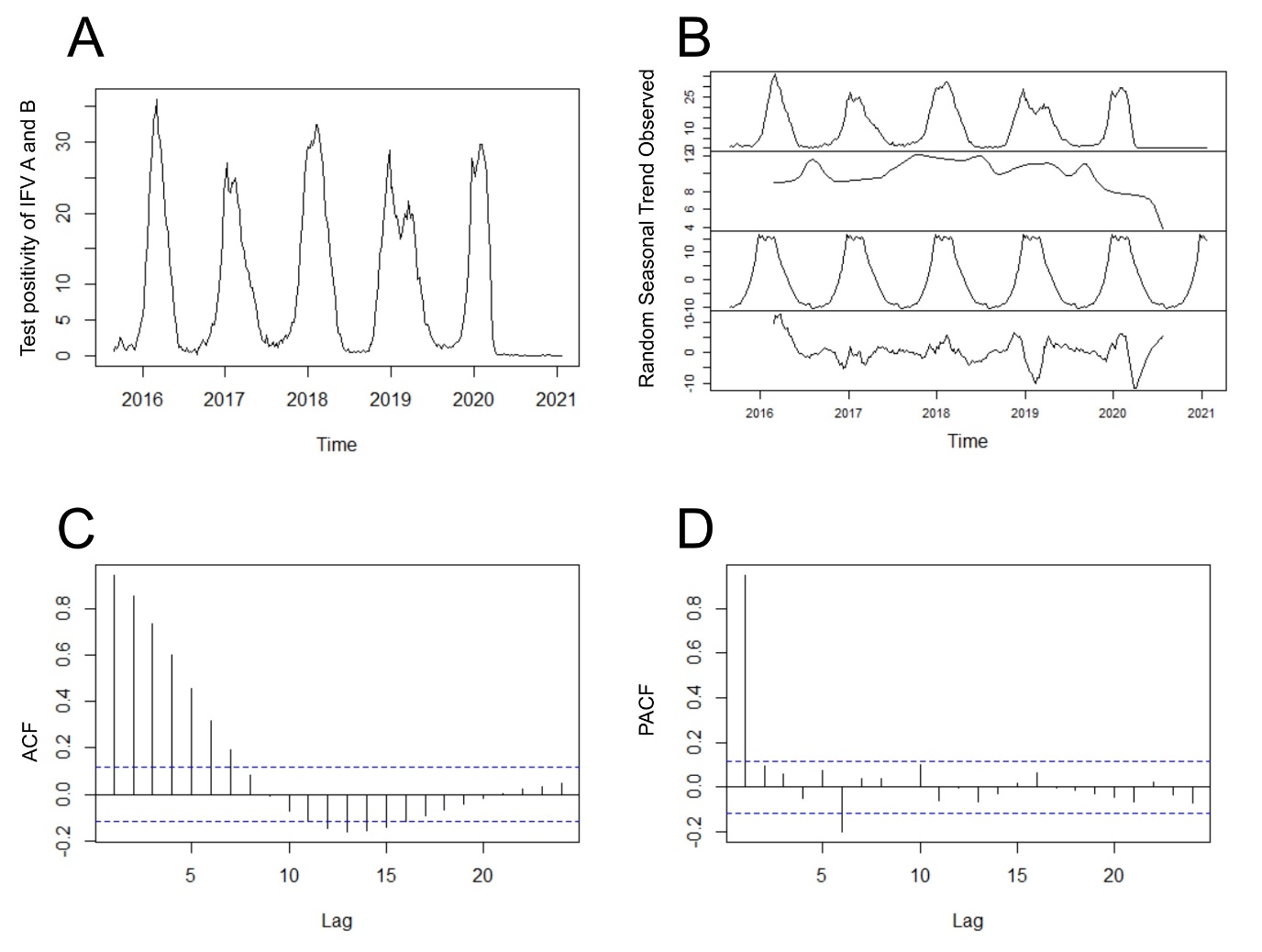


**S4 Table.** Segmented linear regression of influenza A and B viruses test positivity.

|  | ARMA (3,0)* | | ARMA (3,2) | |
| --- | --- | --- | --- | --- |
|  | Coefficient | P-value | Coefficient | P-value |
| Intercept | 9.314 | <0.001 | 9.285 | <0.001 |
| Preslope | 0.006 | 0.585 | 0.007 | 0.522 |
| Intervention | 1.613 | 0.149 | 1.271 | 0.260 |
| Postslope | -0.388 | <0.001 | -0.363 | <0.001 |
| AIC | 919.34 | | 921.28 | |
| BIC | 948.48 | | 957.70 | |

*The final model

**S5 Table.** Weekly reported test positivity of RSV (A), decomposition of the time series of RSV (B), autocorrelation function of adjusted test positivity of RSV (C), and partial autocorrelation of adjusted test positivity of RSV (D).


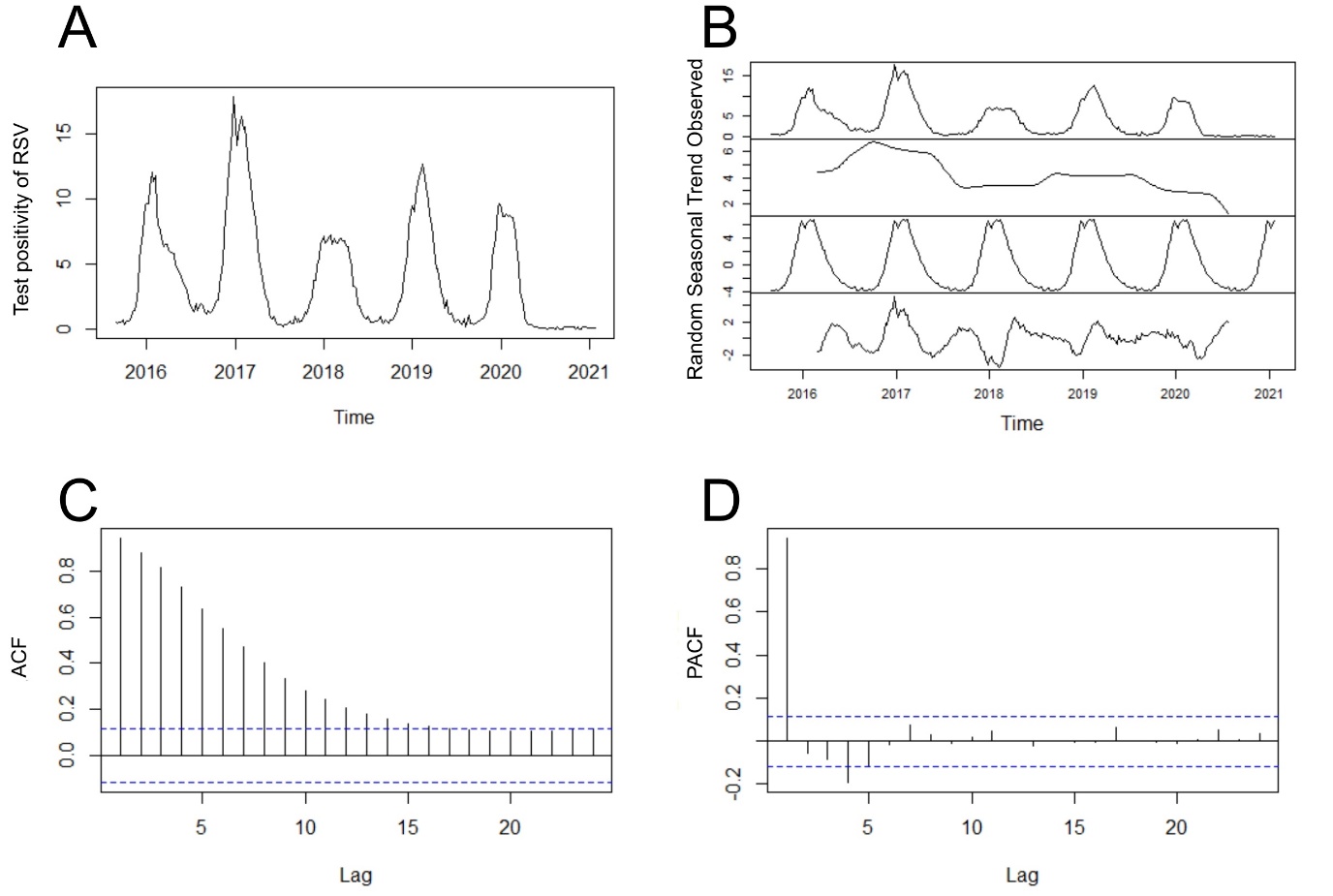


**S6 Table.** Segmented linear regression of RSV test positivity.

|  | ARMA (1,0) | | ARMA (2,1)* | |
| --- | --- | --- | --- | --- |
|  | Coefficient | P-value | Coefficient | P-value |
| Intercept | 4.796 | 0.002 | 5.066 | <0.001 |
| Preslope | -0.003 | 0.770 | -0.005 | 0.313 |
| Intervention | -0.497 | 0.363 | -0.410 | 0.426 |
| Postslope | -0.182 | 0.001 | -0.134 | 0.001 |
| AIC | 457.17 | | 433.16 | |
| BIC | 479.02 | | 462.30 | |

*The final model

**S7 Table.** Weekly reported test positivity of PIV (A), decomposition of the time series of PIV (B), autocorrelation function of adjusted test positivity of PIV (C), and partial autocorrelation of adjusted test positivity of PIV (D).


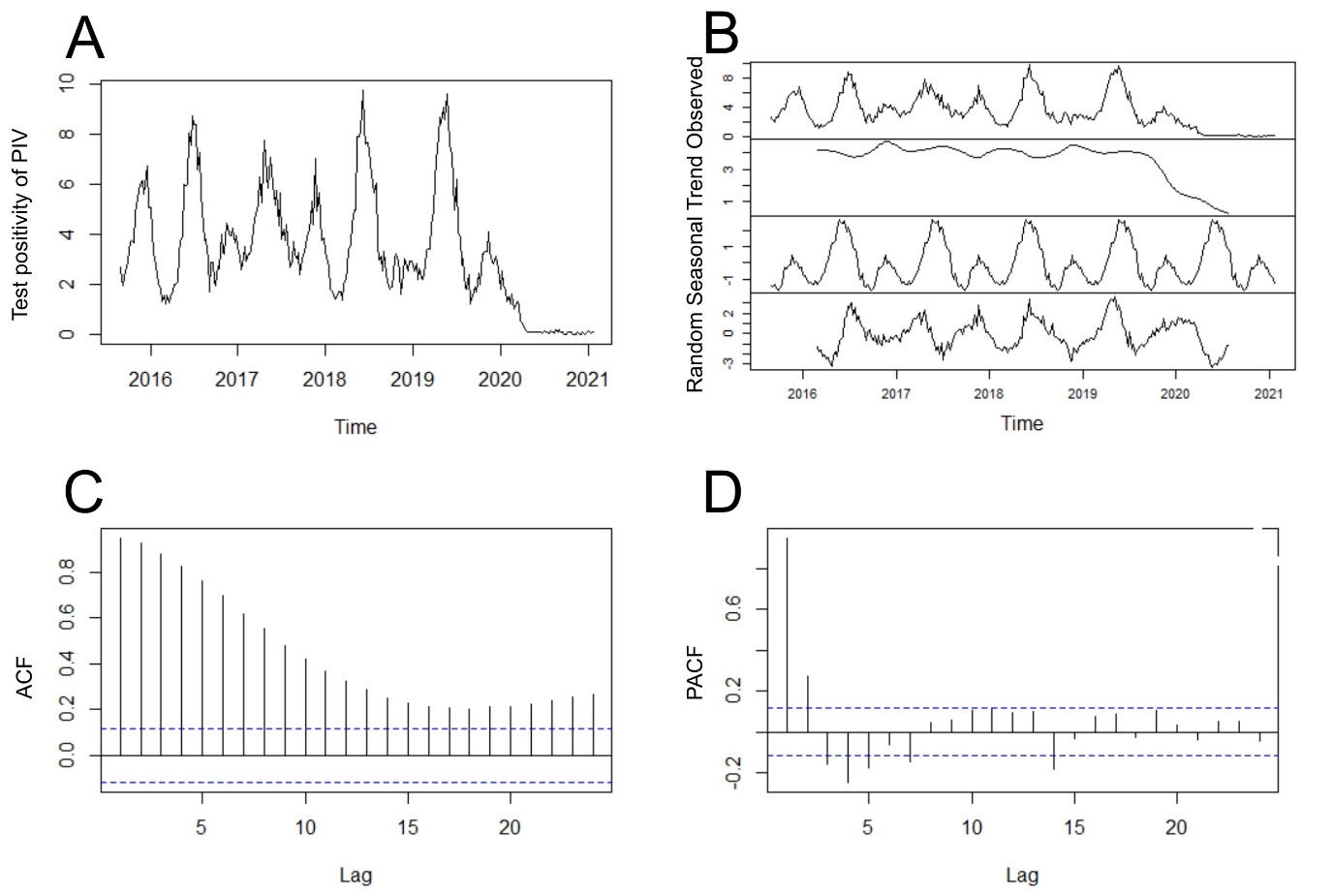


**S8 Table.** Segmented linear regression of PIV test positivity.

|  | ARMA (2,0) | | ARMA (2,2)* | | ARMA (2,3) | |
| --- | --- | --- | --- | --- | --- | --- |
|  | Coefficient | P-value | Coefficient | P-value | Coefficient | P-value |
| Intercept | 4.655 | <0.001 | 4.684 | <0.001 | 4.734 | <0.001 |
| Preslope | -0.008 | 0.268 | -0.007 | 0.062 | -0.008 | 0.200 |
| Intervention | -0.229 | 0.695 | 0.017 | 0.972 | 0.050 | 0.927 |
| Postslope | -0.044 | 0.272 | -0.076 | 0.013 | -0.056 | 0.160 |
| AIC | 523.34 | | 476.55 | | 507.60 | |
| BIC | 548.83 | | 509.33 | | 544.02 | |

*The final model

**S9 Table.** Weekly reported test positivity of ADV (A), decomposition of the time series of ADV (B), autocorrelation function of adjusted test positivity of ADV (C), and partial autocorrelation of adjusted test positivity of ADV (D).


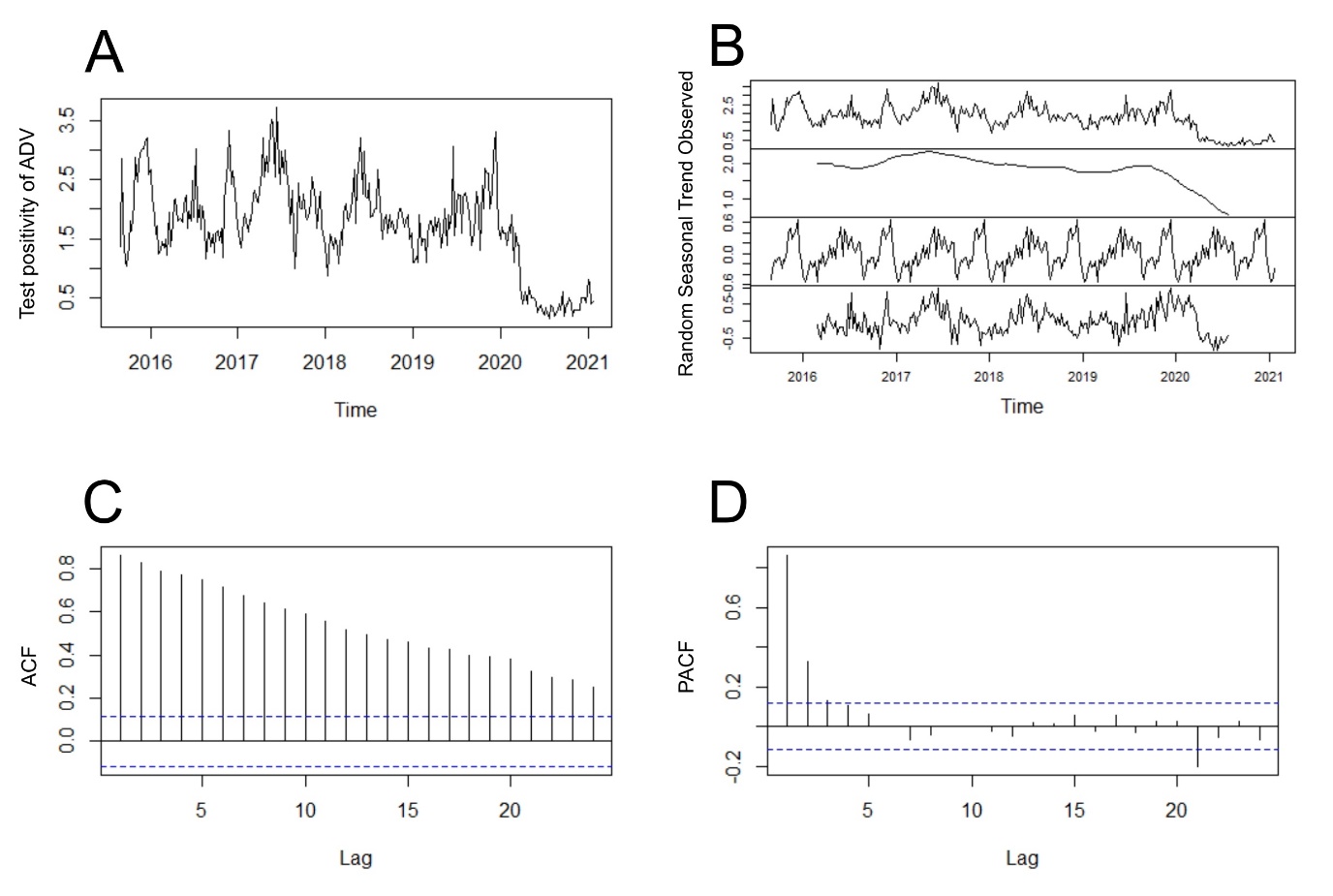


**S10 Table.** Segmented linear regression of ADV test positivity.

|  | ARMA (1,0) | | ARMA (1,1)* | | ARMA (1,2) | |
| --- | --- | --- | --- | --- | --- | --- |
|  | Coefficient | P-value | Coefficient | P-value | Coefficient | P-value |
| Intercept | 2.133 | <0.001 | 2.174 | <0.001 | 2.175 | <0.001 |
| Preslope | -0.001 | 0.104 | -0.002 | 0.159 | -0.002 | 0.155 |
| Intervention | -1.038 | <0.001 | -0.711 | 0.009 | -0.707 | 0.009 |
| Postslope | -0.011 | 0.192 | -0.015 | 0.145 | -0.016 | 0.139 |
| AIC | 204.91 | | 187.12 | | 189.09 | |
| BIC | 226.76 | | 212.61 | | 218.23 | |

*The final model

**S9 Table.** Weekly reported test positivity of MPV (A), decomposition of the time series of MPV (B), autocorrelation function of adjusted test positivity of MPV (C), and partial autocorrelation of adjusted test positivity of MPV (D).


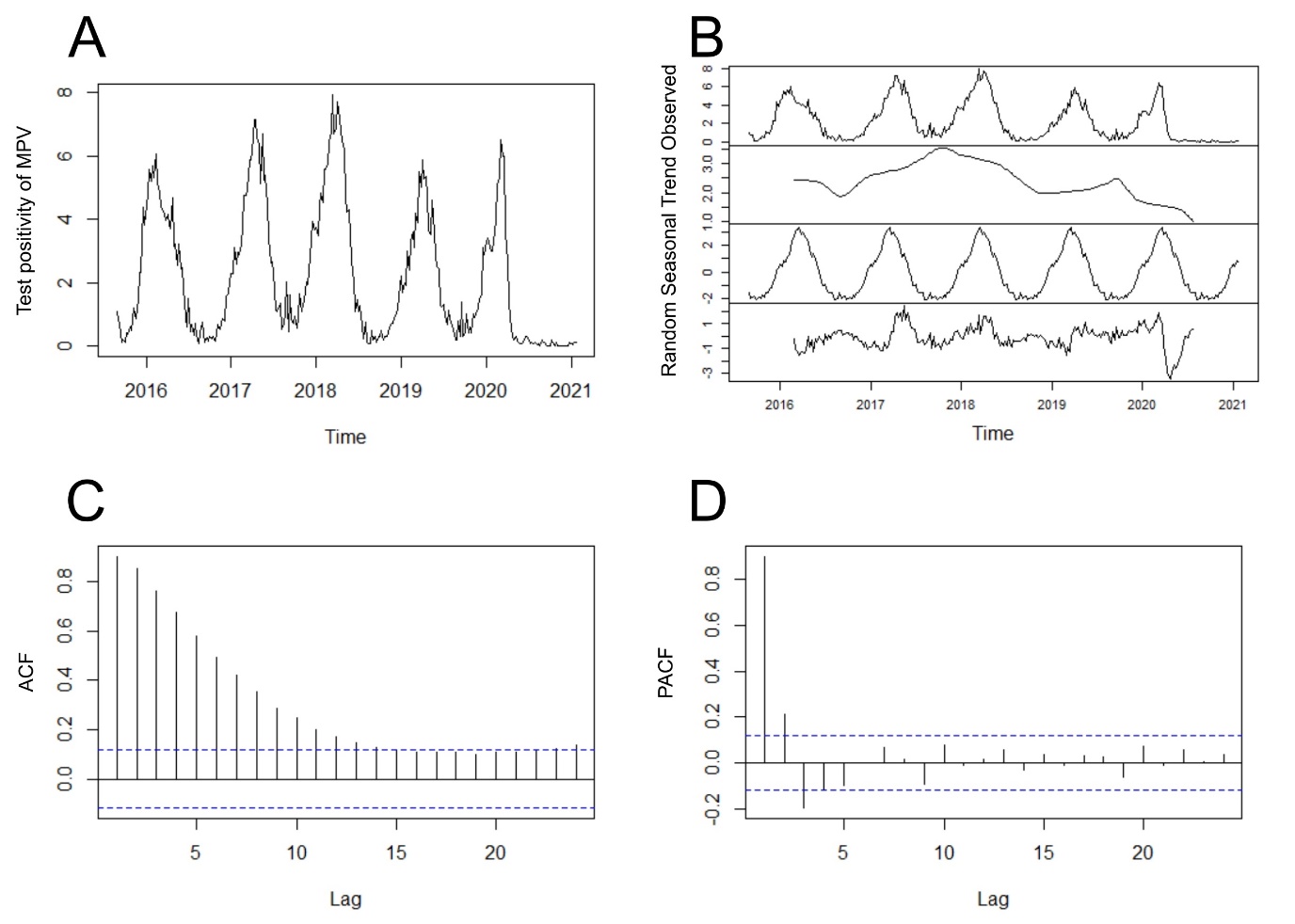


**S10 Table.** Segmented linear regression of MPV test positivity.

|  | ARMA (1,0) | | ARMA (1,2) | | ARMA (2,2)* | |
| --- | --- | --- | --- | --- | --- | --- |
|  | Coefficient | P-value | Coefficient | P-value | Coefficient | P-value |
| Intercept | 2.735 | <0.001 | 2.780 | <0.001 | 2.818 | <0.001 |
| Preslope | -0.002 | 0.635 | -0.002 | 0.569 | -0.003 | 0.444 |
| Intervention | -0.684 | 0.152 | -0.061 | 0.888 | 0.068 | 0.870 |
| Postslope | -0.035 | 0.145 | -0.053 | 0.037 | -0.053 | 0.028 |
| AIC | 394.51 | | 364.22 | | 361.11 | |
| BIC | 416.36 | | 393.35 | | 393.89 | |

*The final model

**S11 Table.** Weekly reported test positivity of RV/EV (A), decomposition of the time series of RV/EV (B), autocorrelation function of adjusted test positivity of RV/EV (C), and partial autocorrelation of adjusted test positivity of RV/EV (D).


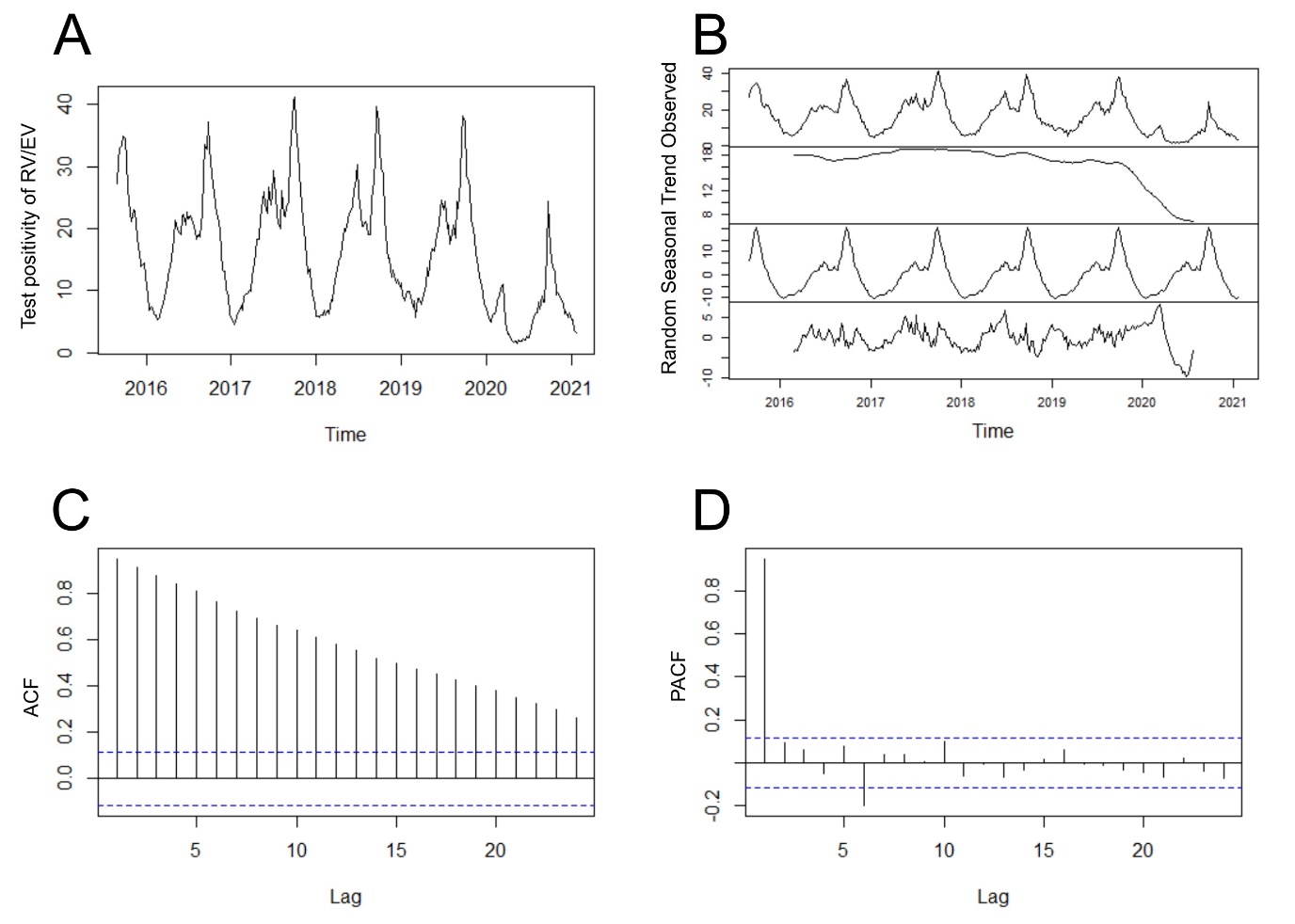


**S12 Table.** Segmented linear regression of RV/EV test positivity.

|  | ARMA (1,0)* | | ARMA (1,1) | | ARMA (1,2) | |
| --- | --- | --- | --- | --- | --- | --- |
|  | Coefficient | P-value | Coefficient | P-value | Coefficient | P-value |
| Intercept | 19.981 | <0.001 | 20.060 | <0.001 | 20.060 | <0.001 |
| Preslope | -0.023 | 0.132 | -0.024 | 0.157 | -0.024 | 0.158 |
| Intervention | -1.440 | 0.414 | -1.396 | 0.424 | -1.401 | 0.422 |
| Postslope | -0.125 | 0.226 | -0.111 | 0.299 | -0.110 | 0.302 |
| AIC | 1121.91 | | 1121.49 | | 1123.491 | |
| BIC | 1143.76 | | 1146.99 | | 1152.626 | |

*The final model

**S13 Table.** Weekly reported test positivity of COV (A), decomposition of the time series of COV (B), autocorrelation function of adjusted test positivity of COV (C), and partial autocorrelation of adjusted test positivity of COV (D).


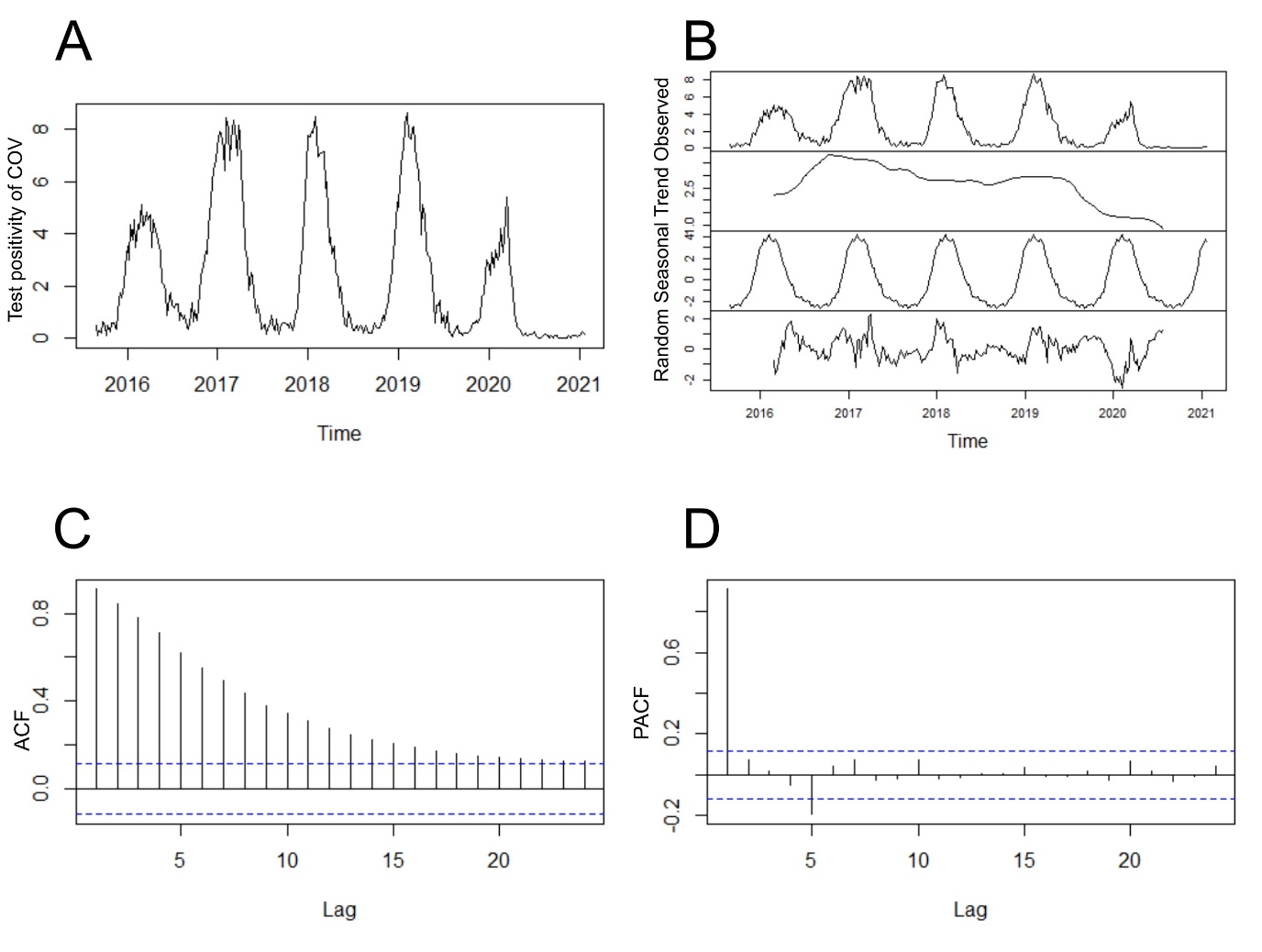


**S14 Table.** Segmented linear regression of COV test positivity.

|  | ARMA (1,0) | | ARMA (1,1)* | | ARMA (1,2) | |
| --- | --- | --- | --- | --- | --- | --- |
|  | Coefficient | P-value | Coefficient | P-value | Coefficient | P-value |
| Intercept | 3.156 | <0.001 | 3.169 | <0.001 | 3.168 | <0.001 |
| preslope | -0.005 | 0.413 | -0.005 | 0.444 | -0.005 | 0.440 |
| intervention | 1.842 | <0.001 | 1.846 | <0.001 | 1.901 | <0.001 |
| postslope | -0.129 | <0.001 | -0.131 | <0.001 | -0.133 | <0.001 |
| AIC | 415.70 | | 409.98 | | 411.04 | |
| BIC | 437.55 | | 435.47 | | 440.18 | |

*The final model
